# AdVance™ male sling for stress urinary incontinence: Long‐term follow‐up and patient satisfaction

**DOI:** 10.1002/bco2.287

**Published:** 2023-09-20

**Authors:** Rebecca Therese Boe, Ole Jacob Nilsen, Henriette Veiby Holm

**Affiliations:** ^1^ Department of Urology Oslo University Hospital Oslo Norway; ^2^ Department of Urology Kristiansund Hospital, Møre og Romsdal Hospital Trust Kristiansund Norway; ^3^ Institute of Clinical Medicine, Faculty of Medicine University of Oslo Oslo Norway

**Keywords:** AdVance XP sling, long‐term follow‐up, male incontinence, male sling, patient satisfaction, postprostatectomy incontinence, PROM, stress urinary incontinence

## Abstract

**Objectives:**

To evaluate long‐term effects, complications and satisfaction among patients treated with AdVance™ and AdVance™ XP slings (AS) at a Norwegian specialist care hospital.

**Materials and Methods:**

Patients who had an AS implanted due to stress urinary incontinence (SUI) 2009–2016 were identified retrospectively. Demographic and perioperative data were extracted from electronic patient files. We did a patient‐reported outcome measure (PROM) survey with the Expanded Prostate Cancer Index Composite (EPIC‐26) urinary domain and a Satisfaction Questionnaire (SQ) 2018–2020. Cure was defined as use of ≤1 pad/day.

**Results:**

The AS was implanted in 165 patients, mainly due to mild to moderate SUI (median leakage 112 g, range 13–589 g/24 h). Preoperative urodynamics showed mild detrusor overactivity (DO) in 11 patients. At 6‐week follow‐up, 148 patients (90%) were cured. The most common complication was urinary retention (*N* = 38), transient in 32 patients (range 1–42 days). Two patients were later operated with division of the sling due to persistent retention. During clinical follow‐up of up to 12 years, 27 patients were reoperated due to persistent/recurrent incontinence. The PROM survey was sent to 125 patients and 115 (92%) replied at median 73 (20–134) months postoperatively. Ninety‐one (79%) used ≤1 pads/day, 97 (85%) were satisfied, one patient‐reported pain. Regression analyses showed that failure (>1 pad/day) was significantly associated with a higher amount of leakage preoperatively and at the 6‐week follow‐up. Total cure rate in the complete cohort was 64% at median 73 (20–134) months follow‐up.

**Conclusions:**

The AS shows good and persistent long‐term results in patients with mild to moderate SUI. The only identified risk factor for long‐term failure was higher amount of leakage preoperatively. The incidence of high body mass index (BMI), DO and previous radiotherapy was low and not significantly associated with failure but is still considered risk factors.

## 1 INTRODUCTION

Urinary incontinence can be incapacitating and lead to reduced health related quality of life.^1^, ^2^ It is a well‐known major long‐term sequela after radical prostatectomy (RP) for prostate cancer and less commonly after benign surgery of the prostate.^3^, ^4^, ^5^ Despite improved RP surgical techniques, postprostatectomy incontinence (PPI) is still a significant problem, although reported incidence rates vary according to definition applied.^6^ During the last two decades, the prevalence of PPI has increased dramatically due to the increased frequency of surgical treatment of prostate cancer and the long life expectancy of those treated.^2^

Continence may improve up to 12 months post RP with conservative treatment, including pelvic floor exercises and lifestyle interventions.^7^, ^8^ When conservative measures fail, further investigation may reveal sphincter insufficiency, bladder dysfunction or other complications after RP, including vesicourethral anastomosis stenosis (VUAS), or any combination of these. Radiation therapy may severely complicate the condition and exclude available surgical treatments.^9^ Treatment needs to be directed to the exact underlying pathology.

To improve continence in case of sphincter insufficiency in men, implantation of the artificial urinary sphincter (AUS) is still considered the gold standard.^10^ In cases of mild to moderate leakage, there are several other surgical techniques to treat male stress urinary incontinence (SUI) available.^11^ In 2006 the AdVance™ male sling (American Medical Systems, Minnetonka, MN, USA) was introduced by Rehder and Gozzi.^12^ The AdVance™ male sling is a retrourethral, transobturator sling of polypropylene mesh. In 2010, the improved version AdVance™ XP was introduced with added directional chevron anchors for enhanced tissue fixation.

We present a long‐term observational study of men treated for mainly mild to moderate SUI, as well as a cross‐sectional long‐term study of the same cohort. The objectives of the study were to evaluate the short and long‐term effects, complications and patient satisfaction with the AdVance™ and AdVance™ XP.

## 2 MATERIALS AND METHODS

### 2.1 Setting and surgical technique

Oslo University Hospital Rikshospitalet is a tertiary referral centre for the treatment of male urinary incontinence. Implantation of AdVance™ male sling was introduced at our department in 2009 as an alternative for patients with mild to moderate SUI.

The indication for AdVance™/AdVance™ XP sling was male SUI. Contraindications included significant bladder dysfunction, nocturnal enuresis and neurogenic lower urinary tract dysfunction. Patients with daily urinary leakage of more than 400 g were usually not offered a sling, with few exceptions. History of pelvic radiation therapy was a relative contraindication during the first part of the study period and an absolute contraindication in the latter part. There was no age limit.

Preoperatively the patients completed voiding diaries and 24‐h pad test for 3 days and underwent clinical examination, urethrocystoscopy and urodynamic evaluations. A detailed patient history was obtained and included patient‐reported residual function of the external sphincter (ability to stop the urinary stream). A preoperative urethrocystoscopy was done to confirm concentric sphincter contraction and the absence of anastomotic or urethral strictures. The urodynamic evaluation was performed primarily to study bladder function, including detrusor activity during both filling and voiding phases, bladder compliance and any sign of obstructive outflow. A prerequisite for sling surgery was absence of detrusor overactivity (DO) during the filling phase (cystometry) and a good detrusor contraction during the pressure flow study. There were no cut‐off limits for these urodynamic findings but the evaluation was at each surgeon's discretion.

Preoperatively all patients had negative urine culture. Surgery was performed as described by Rehder and Gozzi in 2007, in general anaesthesia and with prophylactic antibiotics (cefalotin 2 g iv). A transurethral catheter was routinely left in place for less than 24 h. The patients were discharged the first postoperative day after measurement of post‐void residual (PVR). In case of a high PVR (limit assessed individually), patients were taught clean intermittent self‐catheterization (CISC) or a permanent transurethral/suprapubic catheter was placed. They received extended prophylaxis with cefalexin 500 mg QID for 5 days.

At clinical follow‐up 6 weeks postoperatively, any early complications and continence status was recorded. Most patients did not have further routine follow‐up but were encouraged to return in case of any complications or recurrence of SUI.

### 2.2 Patient sampling—retrospective study

We conducted a study of all patients treated with the AdVance™ or AdVance™ XP male sling at Oslo University Hospital Rikshospitalet during the period of 2009–2016.

The electronic patient files were accessed in 2021 for the retrospective part of this study. Demographic and perioperative data were collected. Preoperative leakage was defined from mild to severe according to amount, with <100 g defined as mild, 100–400 g defined as moderate and >400 g defined as severe. Successful outcome/cure after sling implantation was defined as social continence (0–1 pad/day), and failure was defined as the need for two or more pads/day.

Both early and late complications had been documented in the electronic patient files and were recorded for this study. Early complications assessed included wound infection, urinary tract infection, urinary retention, pain, lower urinary tract symptoms and persistent or recurrent incontinence within the first 6 weeks. Late complications were the same as the abovementioned recorded at any time after the first 6 weeks, as well as any reoperation (division of sling, new sling or implantation of AUS).

### 2.3 PROM survey—cross‐sectional study

**FIGURE 1:**
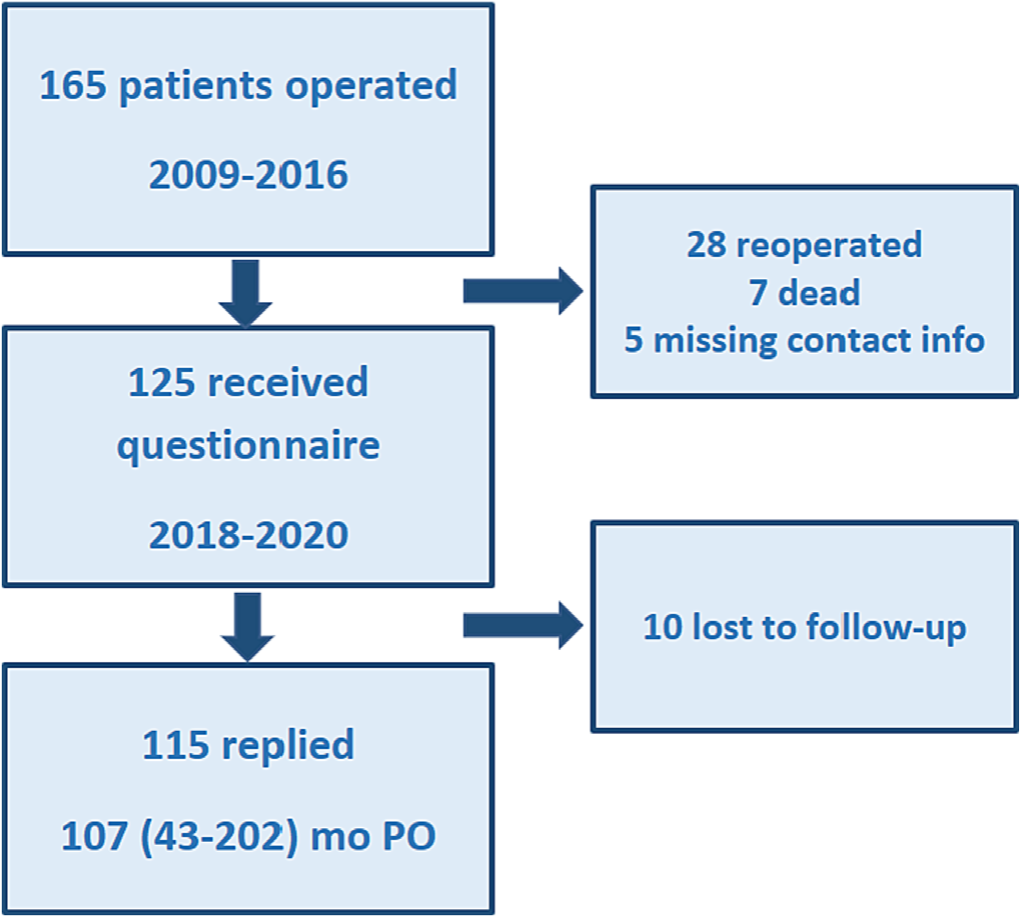
Flow chart of included patients. We included 165 patients, of whom 125 patients were sent questionnaires (PROM survey) during 2018–2020. Patients who had been reoperated after initial sling surgery, were diseased or had missing contact info were not included in the PROM survey. Of the 125 included in the PROM survey, 115 patients replied at a median of 107 months (range 43–202) postoperatively.

A cross‐sectional study with patient‐reported outcome measures (PROMs) for long‐term follow‐up was performed during 2018–2020. Questionnaires were sent to all identified patients operated during the study period 2009–2016 described above, except patients who had later been reoperated, were deceased or in case of missing contact info (Figure 1).

We used the Expanded Prostate Cancer Index Composite (EPIC‐26) score system for urinary assessment and a questionnaire about patients' satisfaction with the incontinence surgery.^13^, ^14^ The *EPIC‐26 questionnaire* concerns self‐rating of urinary functions and sexual, bowel and overall problems after RP. The urinary domain was used for this study (Appendix S1). Definitions of cure and failure were the same as for the retrospective study (0–1 pads/day vs. ≥2 pads/day).

The *Satisfaction Questionnaire* (SQ) rated further aspects of the outcome of the sling surgery, including patient satisfaction, pain and problem with bladder emptying. It has previously been used in a similar study but is not a validated questionnaire (Appendix S2).^15^

### 2.4 Statistical methods

Descriptive statistics were presented using mean and standard deviation (SD) or median and range for continuous variables and frequencies and proportions for categorical variables.

Patients experiencing treatment success were compared to patients experiencing treatment failure using independent *t* tests for continuous variables and Fisher's exact tests for categorical variables. Non‐parametric tests were used if normality could not be assumed.

To investigate associations between treatment success/failure and possible prognostic factors, we estimated a multivariable logistic regression, from which odds ratios (OR) and corresponding confidence intervals (CIs) and *p* values were presented.

Two alternative continuous outcome measures were also defined using EPIC total score and EPIC ‘pad question’. Here, multivariable linear regressions were estimated to identify potential prognostic factors, and regression coefficients and corresponding CIs and *p* values were presented. Potential prognostic factors were identified a priori using a combination of clinical experience and previously published articles on this topic.

Attrition analyses were done to compare baseline characteristics among those who did not respond to the questionnaire (*N* = 15) to those who did respond. Analyses were done using Stata version 17.0.

To calculate the total cure rate in this cohort, we included all patients operated with AS 2009–2016 except those deceased and lost to follow‐up, with unknown long‐term results. Patients reoperated due to incontinence or urinary retention were considered failures.

The level of significance was set at *p* < 0.05, and all tests were two sided.

### 2.5 Ethics

The Ethics Committee for Health Research of the South Eastern Health Region of Norway approved the study (18/01391). Patients returned the questionnaire with written informed consent.

## 3 RESULTS

The AdVance™/AdVance™ XP was implanted in 165 male patients during the study period 2009–2016. The aetiology of SUI was RP (*N* = 155), RP and radiotherapy (*N* = 4) and transurethral resection of the prostate (*N* = 6). After 2011, no patients with a history of radiation were offered AdVance™/AdVance™ XP.

The patients mainly had mild to moderate SUI (pad weight median 112 g/24 h, range 13–589). All but three patients had a preoperative urodynamic evaluation, showing normal bladder function in 151. The remaining 11 patients had mild DO at a median bladder volume of 320 mL (range 200–440). None of the patients had signs of obstruction on urodynamic investigation, and all had a good detrusor contraction during the pressure/flow study. Urethrocystoscopy was performed in all patients, confirming residual sphincter function with concentric contraction and absence of obvious pathology. Detailed baseline characteristics are shown in Table 1.

**TABLE 1:** Baseline characteristics at time of evaluation prior to sling surgery.

Included patients	*N*	165
Index surgery		
TURP	*N* (%)	6 (4)
Radical prostatectomy	*N* (%)	155 (94)
RP and RT	*N* (%)	4 (2)
Time from index surgery to sling surgery (years)	Median (range)	2.7 (0.9–10.8)
Age (years)	Mean (±SD)	66.5 ± 5.9
BMI (kg/m^2^)	Mean (±SD)	26.6 ± 2.9
Amount of leakage (g/24 h)	Median (range)	112 (13–589)
Degree of leakage		
Mild <100 g/24 h	*N* (%)	65 (39)
Moderate 100–400 g/24 h	*N* (%)	79 (48)
Severe >400 g/24 h	*N* (%)	15 (9)
Missing data	*N* (%)	6 (4)
Number of pads/24 h		
1–2 pads	*N* (%)	24 (15)
3–4 pads	*N* (%)	88 (53)
≥5 pads	*N* (%)	41 (25)
Missing data	*N* (%)	12 (7)
Maximum bladder capacity on voiding diaries (mL)	Mean (±SD)	465 ± 117
Qmax on free uroflowmetry (mL/s)	Median (range)	23 (6–70)
Smallest residual volume (mL)	Median (range)	0 (0–138)
Cystometry and pressure/flow study	*N* (%)	162 (98)
Detrusor overactivity present during filling phase	*N* (%)	11 (7)
First contraction at bladder volume (mL)	Median (range)	320 (200–440)
Highest detrusor pressure (cmH_2_O)	Median (range)	30 (15–43)
Urethrocystoscopy confirming normal urethra	*N* (%)	165 (100)

Abbreviations: BMI, body mass index; Qmax, maximum flow rate on free uroflowmetry; RP, radical prostatectomy; RT, radiotherapy; TURP, transurethral resection of prostate.

There were no intraoperative complications. Urinary retention or high PVR was the only complication occurring during the 24‐hour hospital stay, described in more detail below.

### 3.1 Follow‐up 6 weeks postoperatively

At first follow‐up 6 weeks postoperatively, 148 patients (89.7%) were identified as cured and the remaining 17 patients (10.3%) as failures. We found no baseline characteristics that were significantly associated with continence status at the 6‐week postoperative clinical follow‐up.

Seven patients, none of whom had DO at preoperative urodynamic evaluation, reported de novo urgency. Urinary tract infection occurred in two patients (Clavien–Dindo grade 2). There were no wound infections. The most common early complication was unacceptable PVR or urinary retention (*N* = 38, 23.0%). For most of these patients (*N* = 32, 19.4%), the urinary retention was transient (Clavien–Dindo grade 3a). They were managed with indwelling transurethral/suprapubic catheter or CISC for a median of 14 days (range 1–42 days) before regaining normal voiding without significant PVR.

### 3.2 Long‐term clinical follow‐up after 6 weeks

Clinical follow‐up was up to almost 12 years, from the first implantation in 2009 to 2021 when the electronic patient files were accessed. During this period, 36 patients (21.8%) reported recurrent or persistent incontinence during long‐term clinical follow‐up. Six patients (3.6%) had persistent urinary retention and continued CISC. There were no other late complications, such as infections or erosions.

Reoperations for urinary incontinence and/or urinary retention (sling division) were done at a median of 25 months (range 1–111) after primary sling surgery. Of 36 patients who reported incontinence during follow‐up, 26 patients (16.4%) were reoperated with a continence procedure (second sling or AUS), of which three had had transient urinary retention postoperatively. Of the 10 incontinent patients who were not reoperated, four reported de novo urgency and were effectively treated with antimuscarinics.

A second sling was implanted in seven patients, with median leakage of 112 g (range 50–217 g/24 h). An AUS was implanted in 20 patients, with median leakage of 150 g (range 100–200 g/24 h).

Sling division was done in two of the patients with persistent urinary retention (Clavien–Dindo grade 3b). One of these patients had an AUS implanted in the same procedure. The other patient (post‐TURP) was continent after sling division but still had incomplete bladder emptying in need of CISC.

Of the four patients with a history of salvage radiotherapy, two had urinary retention postoperatively, and three were later reoperated with an AUS due to persistent/recurrent incontinence.

Of the six post‐TURP patients, three had urinary retention postoperatively, one of whom had the sling divided. All reported continence and satisfaction at the 6‐week follow‐up and beyond.

### 3.3 PROM survey

In 2018–2020, 125 patients were invited to the PROM survey by filling out questionnaires sent to them by mail (Figure 1). The reply rate was 92% (*N* = 115) at a median of 73 (20–134) months after sling implantation (Table 3). Two thirds of the patients (*N* = 77) had longer follow‐up than 60 months. One questionnaire was incomplete, missing the reply to two questions (EPIC total, satisfaction), and three to five patients did not to reply to page two of the questionnaire (EPIC‐26 Q6). The remaining patients replied to all questions.

The results of the PROM survey are listed in detail in Table 2. Use of no pads was reported by 42% of the patients, use of one pad daily was reported by 37% of the patients, that is, a total of 79% of the patients were defined as socially continent or cured according to our definition. Eighty‐one per cent reported *no* or *small problem* with their urinary function, and 85% reported satisfaction with the result of the sling operation.

**TABLE 2:** Results of the long‐term follow‐up cross‐sectional study (*the PROM survey*).

The Expanded Prostate Cancer Index Composite Urinary assessment: EPIC‐26 urinary domain	
This section is about your urinary habits. Please consider only the last 4 weeks.	
EPIC‐26 Q1. Over the past 4 weeks, how often have you leaked urine?	*N* = 115
More than once a day	43 (37.4%)
About once a day	17 (14.8%)
More than once a week	4 (3.5%)
About once a week	4 (3.5%)
Rarely or never	47 (40.9%)
EPIC‐26 Q4. Which of the following best describes your urinary control during the past 4 weeks?	*N* = 115
No urinary control whatsoever	4 (3.5%)
Frequent dribbling	12 (10.4%)
Occasional dribbling	70 (60.9%)
Total control	29 (25.2%)
EPIC‐26 Q5. How many pads or adult diapers per day did you usually use to control leakage during the last 4 weeks?	*N* = 115
None	48 (41.7%)
1 pad per day	43 (37.4%)
2 pads per day	12 (10.4%)
3 or more pads per day	12 (10.4%)
EPIC‐26 Q6. How big a problem, if any, has each of the following been for you during the last 4 weeks?	
EPIC‐26 Q6 a. Dripping or leaking urine	*N* = 110
No problem	36 (32.7%)
Very small problem	22 (20.0%)
Small problem	24 (21.8%)
Moderate problem	19 (17.3%)
Big problem	9 (8.2%)
EPIC‐26 Q6 d. Weak urine stream or incomplete emptying	*N* = 112
No problem	63 (56.3%)
Very small problem	22 (19.6%)
Small problem	18 (16.1%)
Moderate problem	9 (8.0%)
Big problem	1 (0.9%)
EPIC‐26 Q6 e. Waking up to urinate	*N* = 112
No problem	39 (34.8%)
Very small problem	23 (20.5%)
Small problem	22 (19.6%)
Moderate problem	23 (20.5%)
Big problem	5 (4.5%)
EPIC‐26 Q6 f. Need to urinate frequently during the day	*N* = 112
No problem	47 (42.0%)
Very small problem	21 (18.6%)
Small problem	22 (19.6%)
Moderate problem	21 (18.6%)
Big problem	2 (1.8%)
EPIC‐26 Q7. Overall, how big a problem has your urinary function been for you during the last 4 weeks?	*N* = 114
No problem	44 (38.6%)
Very small problem	26 (22.8%)
Small problem	22 (19.3%)
Moderate problem	13 (11.4%)
Big problem	9 (7.9%)

SQ	
This section is about the outcome of your continence surgery (AdVance sling).	
SQ 1. Are you satisfied with the result of the continence surgery?	*N* = 114
Very satisfied	76 (66.7%)
Somewhat satisfied	21 (18.4%)
Unsure	9 (81.8%)
Unsatisfied	8 (7.0%)
SQ 2. Have you had any problems emptying your bladder the last 4 weeks?	*N* = 114
No	101 (88.6%)
Yes	13 (11.4%)
SQ 3. Have you had any pain due to the continence surgery the last 4 weeks?	*N* = 114
No	113 (99.1%)
Yes	1 (0.9%)
SQ 4. Would you recommend this operation to others with urinary leakage?	*N* = 114
Yes	91 (79.8%)
Yes, but with reservation	12 (10.5%)
Unsure	8 (7.0%)
No	3 (2.6%)
SQ 5. Would you have chosen the same surgery again?	*N* = 114
No	6 (5.3%)
Yes	108 (94.7%)
SQ 6. Has the continence surgery had any influence on your sexual function?	*N* = 113
Yes, it has improved	12 (10.6%)
No, it had no influence on my sexual function	70 (61.9%)
Yes, it worsened	31 (27.4%)

*Note*: The questionnaires are available in their complete versions as Appendices S1 and S2. Some questions were omitted in this table due to little relevance and very low incidence (e.g., EPIC‐26 Q2, EPIC‐26 Q3, EPIC‐26 Q6 b and EPIC‐26 Q6 c concerning haematuria and dysuria). *N* = number of patients replying to each of the questions and equals the denominator calculating the percentage of each reply.

Abbreviations: EPIC, Expanded Prostate Cancer Index Composite Urinary assessment; PROM, patient‐reported outcome measure; SQ, Satisfaction Questionnaire.

Among the six post‐TURP patients, four were included in the PROM survey, of whom three replied; all were satisfied. Of the remaining two, one had been reoperated with sling division due to urinary retention and one was deceased.

Among the 32 patients with transient urinary retention, 26 patients were included in the PROM survey, of whom 23 replied: 21 reported satisfaction and two dissatisfaction.

To calculate the total cure rate of this cohort operated 2009–2016, at the time of the PROM survey (median follow‐up 73 months, range 20–134), we included the 115 patients who replied to the PROM survey (91 cured, 24 failures, Table 2) as well as those already defined as failures at that time (28 reoperated, not included in the PROM survey, Figure 1). Hence, we identified 91 cured patients and 52 failures (total 143) during the follow‐up, that is, the total cure rate was 63.6%. Of the remaining 22 patients, seven are deceased and 15 have an unknown long‐term result. An attrition analysis of these 15 lost to follow‐up showed no baseline differences to those included in this analysis.

The PROM survey analyses revealed that higher (i.e., better) EPIC urinary domain total score was associated with lower age at sling implantation (*p* = 0.032), lesser preoperative leakage (g/24 h) (*p* = 0.044) and better continence status at 6 weeks (*p* = 0.001) (Table 3). Higher score on Question 5 of the EPIC‐26 (i.e., less pad use) was associated with less preoperative leakage (g/24 h) (*p* = 0.000) and better continence status at 6 weeks (*p* = 0.009).

**TABLE 3:** Risk factor analysis based on the PROM survey.

	Outcome: EPIC‐26 Q5 (number of pads/day)		Outcome: EPIC‐26 urinary domain total score	
	Coefficient (95% CI)	*p* value	Coefficient (95% CI)	*p* value
Preoperative leakage (g/24 h)	−0.08 (−0.13 to −0.04)	**<0.001**	−0.02 (−0.05 to −0.00)	**0.044**
Stable bladder on cystometry (ref)	1		1	
Detrusor overactivity	−1.01 (−23.37 to 21.36)	0.929	−6.91 (−18.37 to 4.55)	0.234
Age at surgery	−0.34 (−1.40 to 0.72)	0.524	−0.59 (−1.14 to 0.05)	**0.032**
BMI at surgery	0.18 (−1.78 to 2.13)	0.858	−0.14 (−1.15 to 0.86)	0.775
Continent at 6 weeks (ref)	1		1	
Incontinent at 6 weeks	−18.87 (−32.01 to −5.74)	**0.005**	−11.83 (−18.56 to 5.09)	**0.001**
Years from sling implant to PROM survey	−1.65 (−4.46 to 1.17)	0.248	−0.99 (−2.43 to 0.45)	0.177

*Note*: Two continuous outcome measures were defined using EPIC‐26 urinary domain total score and EPIC‐26 Q5 (number of pads used daily). Here, multivariable linear regressions were estimated to identify potential prognostic factors, and regression coefficients and corresponding CIs and *p* values were presented. Potential prognostic factors were identified a priori using a combination of clinical experience and previously published articles on this topic. Numbers in bold indicate statistical significance (*p* < 0.05).

Abbreviations: BMI, body mass index; CI, confidence interval; EPIC‐26 Q5, Question 5 of the questionnaire (number of pads used daily); EPIC‐26, Expanded Prostate Cancer Index Composite; PROM, patient‐reported outcome measure.

Of the four patients with a history of pelvic radiotherapy, three were failures; however, this information could not be included in multivariable analyses due to the low number of patients.

## 4 DISCUSSION

In this cohort, the cure rate with the AdVance™ and AdVance™ XP sling was 63.6% at long‐term follow‐up of a median of more than 6 years. The most common postoperative complication was transient urinary retention. The main indication for reoperation was recurring incontinence. Among those not reoperated, 79% were still socially continent and 85% were satisfied up to more than 11 years after sling implantation. Failure of long‐term continence was associated with more leakage preoperatively and at the 6‐week follow‐up.

Some known risk factors, like obesity and previous radiation, could not be identified as risk factors in this study. However, these characteristics were rare in our cohort and may have been missed as risk factors due to the small numbers. Patients with DO did not report statistically significant worse outcome than those without DO regarding continence, lower urinary tract symptoms or complications postoperatively, which may also be due to the low incidence and low grade of severity of this feature in our cohort.

The definition of cure after sling implantation varies in the literature, from pad free to 0–1 pad daily or up to 5 g/24 h. The latter two definitions (≤1 pad or ≤5 g daily) are considered interchangeable. Those using the definition *pad free* have shown cure rates of 67% at 49 months median FU (*N* = 94)^16^ and 62% at 21 months mean FU (*N* = 136).^17^ Reported cure rates with the definition 0–1 *dry* pad are 51% at 52 months median FU (*N* = 72) and 53% at 36 months FU (*N* = 151).^18^, ^19^ Authors using the definition 0–1 pads^20^ have reported a cure rate of 66% at 5 years FU (*N* = 172) while those using the definition leakage of up to 5 g/24 h^21^, ^22^, ^23^, ^24^ have shown cure rates of 46% to 72% at up to 5 years FU (*N* = 15–115).

A systematic review and meta‐analysis of male slings from 2019 highlights this issue of different definitions of cure and continence, the most commonly used being ‘social continence’ (*0–1 pad or minimal leakage in pad test*).^25^ Cure rates with AdVance™/AdVance™ XP vary from 9% to 86.9% (20 studies, with FU 12–61.5 months). Cure rates with other slings (InVance, TONS, I‐Stop TOMS, Surgimesh M‐sling, TiLoop, Virtue, Argus/ArgusT, ATOMS, Remeex, 18 studies, with FU 12–58 months) vary from 15% to 90%.^25^

Hence, the most common definition of cure after treatment of male SUI with a sling is so‐called social continence (0–1 pad/day or ≤5 g/24 h). Using this definition, our results are comparable with previously published data (64% vs. 9% to 86.9%), especially considering the longer follow‐up and this heterogenic population.

An article from 2021^26^ describes which definition of pad usage that best reflects patients' perception of quality of life, where the use of no pad was associated with better quality of life than using one safety pad or one pad. This may lead us to reconsider the definition of cure in future studies and rather use the definition pad free as a successful outcome.

As in most other studies of the AdVance™/AdVance™ XP slings, patients were carefully selected for this method: Patients with nocturnal incontinence, previous radiotherapy and a coaptive zone of <1 cm are usually not offered a sling.^27^ All our patients had a preoperative urethrocystoscopy to verify a healthy urethra and a concentric contraction of the sphincter, though evaluation of the coaptation zone with the repositioning test has not been systematically recorded in our patient files.

We follow largely the same criteria for recommending AdVance™ sling as reported in other studies. The practice of offering AdVance™ sling to previously irradiated patients was abandoned at our institution in 2011. Contemporary exclusion criteria are previous radiation therapy, severe urinary leakage, nocturnal enuresis and urgency urinary incontinence. Furthermore, the patients should have a normal urethrocystoscopy, good bladder capacity and function and some residual sphincter function.

Regarding the AdVance™ and AdVance™ XP male slings, no difference in success rates have been reported in the literature, but one study reports urinary retention rates of 3.7% and 10.3%, respectively.^28^ In our study, there are too few patients to compare success rates and complication rates between the two slings, so all patients are analysed together.

Previously published studies have indicated low risk of complications after surgery with the AdVance™/AdVance™ XP.^21^, ^23^ Problems like urinary retention (up to 67.3%), pain (up to 1.5%) and de novo urgency are reported to varying degrees at long‐term follow‐up.^25^ In our cohort, urinary retention was quite common but transient for most, and at long‐term follow‐up (PROM survey), most of these patients reported satisfaction. Potential complications should nevertheless be addressed at preoperative counselling with patients. No risk factor for urinary retention has been identified,^29^ but we are careful with offering slings to patients with a low bladder contractility index.

In patients without known risk factors like previous radiotherapy, that is, index patients, adjustable slings might lead to better continence results but are also associated with higher a risk of complications.^25^

The slings have the advantage that it has no mechanical components like the AUS. The risk of complications to an AUS is not negligible and includes infection, erosion and mechanical failure. Patients prefer to avoid a mechanical device and will therefore often choose a male sling over an AUS, if given the choice.^30^ Patients of higher age and with more severe leakage should be preoperatively counselled about a higher risk of persistent incontinence and lower urinary tract symptoms after sling surgery. There is a place for the male sling in the treatment of male SUI, but the AUS remains the best option for patients with severe leakage and/or concomitant bladder dysfunction.

There are limitations to our study. This is a retrospective single centre study of results after surgery performed by experienced surgeons. The patient selection was strict due to the recommendation of previous studies, changing somewhat during the study period. After a few patients in the early phase, we no longer offer the AS to patients with SUI after TURP or a history of pelvic radiation. The long‐term cross‐sectional study with questionnaires used both validated and non‐validated PROMs, the latter being a limitation.

## 5 CONCLUSION

The AdVance™/AdVance™ XP show good and persistent long‐term results in patients with mild to moderate SUI with a follow‐up of up to 134 months. There was no evidence of a decrease of efficacy over the years. More leakage preoperatively was associated with long‐term failure. The incidence of high BMI, mild DO and previous pelvic radiotherapy was low in this cohort and was not significantly associated with failure but is still considered risk factors.

## AUTHOR CONTRIBUTIONS

Alexander Schultz, Henriette Veiby Holm and Ole Jacob Nilsen conceived and designed the research. Rebecca Therese Boe and Henriette Veiby Holm analysed the data and wrote the original draft. Rebecca Therese Boe, Henriette Veiby Holm and Ole Jacob Nilsen reviewed and edited the manuscript. Dr. Alexander Schultz passed away unexpectedly before the results were finalized. All authors have read and approved the submitted version of the manuscript.

## CONFLICT OF INTEREST STATEMENT

R.T.B. reports no conflicts of interest. O.J.N. reports personal honoraria for lectures from Astellas and stocks in Bio Medical Device AS. H.V.H. reports personal honoraria for lectures from Astellas and travel support from Mediq Norge.

## Supporting information


**Appendix S1.** Supporting Information.

**Appendix S2.** Supporting Information.

## References

[1] Coyne KS , Kvasz M , Ireland AM , Milsom I , Kopp ZS , Chapple CR . Urinary incontinence and its relationship to mental health and health‐related quality of life in men and women in Sweden, the United Kingdom, and the United States. Eur Urol. 2012;61(1):88–95. 10.1016/j.eururo.2011.07.049 21831517

[2] Sanda MG , Dunn RL , Michalski J , Sandler HM , Northouse L , Hembroff L , et al. Quality of life and satisfaction with outcome among prostate cancer survivors. N Engl J Med. 2008;358(12):1250–1261. 10.1056/NEJMoa074311 18354103

[3] Haglind E , Carlsson S , Stranne J , Wallerstedt A , Wilderäng U , Thorsteinsdottir T , et al. Urinary incontinence and erectile dysfunction after robotic versus open radical prostatectomy: a prospective, controlled, nonrandomised trial. Eur Urol. 2015;68(2):216–225. 10.1016/j.eururo.2015.02.029 25770484

[4] Ficarra V , Novara G , Rosen RC , Artibani W , Carroll PR , Costello A , et al. Systematic review and meta‐analysis of studies reporting urinary continence recovery after robot‐assisted radical prostatectomy. Eur Urol. 2012;62(3):405–417. 10.1016/j.eururo.2012.05.045 22749852

[5] Carlsson S , Nilsson AE , Schumacher MC , Jonsson MN , Volz DS , Steineck G , et al. Surgery‐related complications in 1253 robot‐assisted and 485 open retropubic radical prostatectomies at the Karolinska University Hospital, Sweden. Urology. 2010;75(5):1092–1097. 10.1016/j.urology.2009.09.075 20022085

[6] Schröder A , et al. Guidelines on urinary incontinence. In: Arnheim AG , editorEAU guidelines Arnheim, The Netherlands: EAU; 2010. p. 11–28.

[7] Fernández RA , García‐Hermoso A , Solera‐Martínez M , Correa MT , Morales AF , Martínez‐Vizcaíno V . Improvement of continence rate with pelvic floor muscle training post‐prostatectomy: a meta‐analysis of randomized controlled trials. Urol Int. 2015;94(2):125–132. 10.1159/000368618 25427689

[8] Galli S , Simonato A , Bozzola A , Gregori A , Lissiani A , Scaburri A , et al. Oncologic outcome and contience recovery after laparoscopic radical prostatectomy: 3 years' follow‐up in a “second generation center”. Eur Urol. 2006;49(5):859–865. 10.1016/j.eururo.2006.01.035 16519991

[9] Martins FE , Holm HV , Lumen N . Devastated bladder outlet in pelvic cancer survivors: issues on surgical reconstruction and quality of life. J Clin Med. 2021;10(21):4920. 10.3390/jcm10214920 34768438 PMC8584541

[10] Herschhorn S . The artificial urinary sphincter is the treatment of choice for post‐radical prostatectomy incontinence. Can Urol Assoc J. 2008;2(5):536–539. 10.5489/cuaj.924 18953453 PMC2572249

[11] Gacci M , Sakalis V , Karavitakis M , Cornu JN , Gratzke C , Herrmann TRW , et al. European Association of Urology guidelines on male urinary incontinence. Eur Urol. 2022;82(4):387–398. 10.1016/j.eururo.2022.05.012 35697561

[12] Rehder P , Gozzi C . Transobturator sling suspension for male urinary incontinence including post‐radical prostatectomy. Eur Urol. 2007;52(3):860–866. 10.1016/j.eururo.2007.01.110 17316969

[13] Einstein DJ , Patil D , Chipman J , Regan MM , Davis K , Crociani CM , et al. Expanded Prostate Cancer Index Composite‐26 (EPIC‐26) Online: validation of an internet‐based instrument for assessment of health‐related quality of life after treatment for localized prostate cancer. Urology. 2019;127:53–60. 10.1016/j.urology.2019.02.004 30790648

[14] Holm HV , Fosså SD , Hedlund H , Dahl AA . Study of generic quality of life in patients operated for post‐prostatectomy incontinence. J Urol. 2013;20(9):889–895. 10.1111/iju.12077 23418855

[15] Holm HV , Fosså SD , Hedlund H , Schultz A , Dahl AA . How should continence and incontinence after radical prostatectomy be evaluated? A prospective study of patient ratings and changes with time. J Urol. 2014;192(4):1155–1161. 10.1016/j.juro.2014.03.113 24727062

[16] Collado A , Domínguez‐Escrig J , Ortiz Rodríguez IM , Ramirez‐Backhaus M , Rodríguez Torreblanca C , Rubio‐Briones J . Functional follow‐up after Advance® and Advance XP® male sling surgery: assessment of predictive factors. World J Urol. 2019;37(1):195–200. 10.1007/s00345-018-2357-9 29948042

[17] Cornu JN , Sèbe P , Ciofu C , Peyrat L , Cussenot O , Haab F . Mid‐term evaluation of the transobturator male sling for post‐prostatectomy incontinence: focus on prognostic factors. Br J Urol. 2011;108(2):236–240. 10.1111/j.1464-410X.2010.09765.x 20955265

[18] Rehder P , Haab F , Cornu JN , Gozzi C , Bauer RM . Treatment of postprostatectomy male urinary incontinence with the transobturator retroluminal repositioning sling suspension: 3‐year follow‐up. Eur Urol. 2012;62(1):140–145. 10.1016/j.eururo.2012.02.038 22386196

[19] Papachristos A , Mann S , Talbot K , Moon D . AdVance male urethral sling: medium‐term results in an Australian cohort. ANZ J Surg. 2018;88(3):E178–E182. 10.1111/ans.13890 28239989

[20] Del Favero L , Tasso G , Deruyver Y , Tutolo M , Beels E , Schillebeeckx C , et al. Long‐term functional outcomes and patient satisfaction after AdVance and AdVanceXP male sling surgery. Eur Urol Focus. 2022;8(5):1408–1414. 10.1016/j.euf.2022.01.017 35151614

[21] Mumm J‐N , Klehr B , Rodler S , Kretschmer A , Vilsmaier T , Westhofen T , et al. Five‐year results of a prospective multicenter trial: AdVance XP for postprostatectomy‐incontinence in patients with favorable prognostic factors. Urol Int. 2021;105(5‐6):421–427. 10.1159/000512881 33517334

[22] Bauer RM , Grabbert MT , Klehr B , Gebhartl P , Gozzi C , Homberg R , et al. 36‐month data for the AdVance XP® male sling: results of a prospective multicentre study. BJU Int. 2017;119(4):626–630. 10.1111/bju.13704 27862836

[23] Grabbert M , Mumm JN , Klehr B , Kretschmer A , Gebhartl P , Gozzi C , et al. Extended follow‐up of the AdVance XP male sling in the treatment of male urinary stress incontinence after 48 months: results of a prospective and multicenter study. Neurourol Urodyn. 2019;38(7):1973–1978. 10.1002/nau.24101 31297894

[24] Kretschmer A , Buchner A , Leitl B , Grabbert M , Sommer A , Khoder W , et al. Long‐term outcome of the retrourethral transobturator male sling after transurethral resection of the prostate. Int Neurourol J. 2016;20(4):335–341. 10.5213/inj.1632648.324 28043113 PMC5209579

[25] Meisterhofer K , Herzog S , Strini KA , Sebastianelli L , Bauer R , Dalpiaz O . Male slings for postprostatectomy incontinence: a systematic review and meta‐analysis. Eur Urol Focus. 2020;6(3):575–592. 10.1016/j.euf.2019.01.008 30718160

[26] García Cortés Á , Colombás Vives J , Gutiérrez Castañé C , Chiva San Román S , Doménech López P , Ancizu Marckert FJ , et al. What is the impact of post‐radical prostatectomy urinary incontinence on everyday quality of life? Linking Pad usage and International Concultation on Incontinence Quetionnaire Short‐Form (ICIQ‐SF) for a COMBined definition (PICOMB definition). Neurourol Urodyn. 2021;40(3):840–847. 10.1002/nau.24631 33604977

[27] Bauer RM , Gozzi C , Roosen A , Khoder W , Trottmann M , Waidelich R , et al. Impact of the ‘repositioning test’ on postoperative outcome of retroluminar transobturator male sling implantation. Urol Int. 2013;90(3):334–338. 10.1159/000347123 23485964

[28] Hüsch T , Kretschmer A , Thomsen F , Kronlachner D , Kurosch M , Obaje A , et al. The AdVance and AdVance XP male sling in urinary incontinence: is there a difference? World J Urol. 2018;36(10):1657–1662. 10.1007/s00345-018-2316-5 29728764

[29] Zheng Y , Major N , Silverii H , Rac G , Rolef J , Rittenberg L , et al. Urinary retention after AdVance™ Sling: a multi‐institutional retrospective study. Neurourol Urodyn. 2021;40(1):515–521. 10.1002/nau.24591 33348444

[30] Kumar A , Litt ER , Ballert KN , Nitti VW . Artificial urinary sphincter versus male sling for post‐prostatectomy incontinence—what do patients choose? J Urol. 2009;181(3):1231–1235. 10.1016/j.juro.2008.11.022 19152937

