# Supplementary material for: AdVance™ male sling for stress urinary incontinence: Long‐term follow‐up and patient satisfaction
Source: BJUI Compass. 2023 Sep 20;5(1):42–51. doi: 10.1002/bco2.287 (PMC10764177; doi:10.1002/bco2.287)
Supplement: Supplementary file 1 — Appendix S1. Supporting Information. [file BCO2-5-42-s002.pdf]

EPIC

The Expanded Prostate Cancer Index Composite

Urinary Assessment

This questionnaire is designed to measure Quality of Life issues in patients with Prostate cancer. To help us get the most accurate measurement, it is important that you answer all questions honestly and completely.

Remember, as with all medical records, information contained within this survey will remain strictly confidential.

Today's Date (please enter date when survey completed): Month________Day________Year________

Name (optional): ____

Date of Birth (optional): Month__________Day___________Year__________

Copyright 2002. The University of Michigan. All rights reserved.

Do Not Mark in This Space

URINARY FUNCTION

This section is about your urinary habits. Please consider **ONLY THE LAST 4 WEEKS**.

1. Over the **past 4 weeks**, how often have you leaked urine?

   More than once a day..................... 1
   About once a day............................ 2
   More than once a week................... 3 (Circle one number) 23/
   About once a week..........................4
   Rarely or never............................... 5

2. Over the **past 4 weeks**, how often have you urinated blood?

   More than once a day..................... 1
   About once a day............................ 2
   More than once a week................... 3 (Circle one number) 24/
   About once a week..........................4
   Rarely or never............................... 5

3. Over the **past 4 weeks**, how often have you had pain or burning with urination?

   More than once a day..................... 1
   About once a day............................ 2
   More than once a week................... 3 (Circle one number) 25/
   About once a week..........................4
   Rarely or never............................... 5

4. Which of the following best describes your urinary control **during the last 4 weeks**?

   No urinary control whatsoever........... 1
   Frequent dribbling.. 2 (Circle one number) 26/
   Occasional dribbling... 3
   Total control.. 4

Copyright 2002. The University of Michigan. All rights reserved.

Do Not Mark in This Space

5. How many pads or adult diapers per day did you usually use to control leakage **during the last 4 weeks**?

None 0
1 pad per day.. 1
2 pads per day.. 2 (Circle one number)
3 or more pads per day... 3 27/

6. How big a problem, if any, has each of the following been for you **during the last 4 weeks**?

(Circle one number on each line)

	No Problem	Very Small Problem	Small Problem	Moderate Problem	Big Problem	
a. Dripping or leaking urine	0	1	2	3	4	28/
b. Pain or burning on urination.....	0	1	2	3	4	29/
c. Bleeding with urination............	0	1	2	3	4	30/
d. Weak urine stream or incomplete emptying………..	0	1	2	3	4	31/
e. Waking up to urinate…………..	0	1	2	3	4	32/
f. Need to urinate frequently during the day …………………………..	0	1	2	3	4	33/

7. Overall, how big a problem has your urinary function been for you **during the last 4 weeks**?

No problem.................................... 1
Very small problem......................... 2
Small problem.................................3 (Circle one number) 34/
Moderate problem.......................... 4
Big problem.................................... 5

THANK YOU VERY MUCH!!

Copyright 2002. The University of Michigan. All rights reserved.
