# Supplementary material for: AdVance™ male sling for stress urinary incontinence: Long‐term follow‐up and patient satisfaction
Source: BJUI Compass. 2023 Sep 20;5(1):42–51. doi: 10.1002/bco2.287 (PMC10764177; doi:10.1002/bco2.287)
Supplement: Supplementary file 2 — Appendix S2. Supporting Information. [file BCO2-5-42-s001.pdf]

SQ Satisfaction Questionnaire

This section is about the outcome of your continence surgery (AdVance sling).

SQ 1. Are you satisfied with the result of the continence surgery?

- □ Very satisfied
- □ Somewhat satisfied
- □ Unsure
- □ Unsatisfied

SQ 2. Have you had any problems emptying your bladder the last four weeks?

- □ No
- □ Yes

SQ 3. Have you had any pain due to the continence surgery the last four weeks?

- □ No
- □ Yes

SQ 4. Would you recommend this operation to others with urinary leakage?

- □ Yes
- □ Yes, but with reservation
- □ Unsure
- □ No

SQ 5. Would you have chosen the same surgery again?

- □ No
- □ Yes

SQ 6. Has the continence surgery had any influence on your sexual function?

- □ Yes, it has improved
- □ No, it had no influence on my sexual function
- □ Yes, it worsened
